# Intradiscal Injection of Adinizer-Processed Minimally Microfragmented Adipose Tissue for Degenerative Lumbar Spondylolisthesis: A Case Report With a Two-Year Follow-Up

**DOI:** 10.7759/cureus.107584

**Published:** 2026-04-23

**Authors:** Yonghyun Yoon, Ji Hyo Hwang, Jaeyoung Lee, Jaewoo Lim, Teinny Suryadi, Anwar Suhaimi, Jonghyeok Lee, Seungbeom Kim, King Hei Stanley Lam

**Affiliations:** 1 Department of Orthopedics, International Academy of Musculoskeletal Medicine, Hong Kong, HKG; 2 Department of Orthopedics, International Academy of Regenerative Medicine, Incheon, KOR; 3 Department of Orthopedics, MSKUS, San Diego, USA; 4 Department of Orthopedic Surgery, Hallym University Kangnam Sacred Heart Hospital, Seoul, KOR; 5 Department of Orthopedic Surgery, Incheon Terminal Orthopedic Surgery Clinic, Incheon, KOR; 6 Department of Physical Medicine and Rehabilitation, Medistra Hospital, Jakarta Pusat, IDN; 7 Department of Physical Medicine and Rehabilitation, Synergy Clinic, Jakarta, IDN; 8 Department of Physical Medicine and Rehabilitation, Hermina Podomoro Hospital, Jakarta, IDN; 9 Department of Rehabilitation Medicine, University Malaya Medical Centre, Kuala Lumpur, MYS; 10 Department of Neurosurgery, Bareun Neurosurgery Clinic, Cheongju-si, KOR; 11 Department of Neurosurgery Pain Medicine, Miso Pain Clinic, Suwon, KOR; 12 Faculty of Medicine, The Chinese University of Hong Kong, Hong Kong, HKG; 13 Faculty of Medicine, The University of Hong Kong, Hong Kong, HKG; 14 The Board of Clinical Research, The Hong Kong Institute of Musculoskeletal Medicine, Hong Kong, HKG

**Keywords:** adinizer, chronic low back pain, degenerative lumbar spondylolisthesis, intradiscal injection, microfragmented adipose tissue, minimally manipulated adipose tissue

## Abstract

Degenerative lumbar spondylolisthesis is a common cause of chronic low back pain and functional limitation in older adults. Although conservative treatment is generally considered first-line management, some patients continue to experience persistent symptoms, and surgery may not always be feasible or acceptable. We report the two-year clinical course of a 72-year-old woman with symptomatic degenerative lumbar spondylolisthesis who underwent intradiscal injection of Adinizer-processed minimally microfragmented adipose tissue (MFAT). She had longstanding chronic low back pain for approximately six years, with noticeable worsening over the preceding six months, marked morning stiffness, pain aggravated by lumbar extension, and bilateral buttock pain. Lumbar radiographs showed less than 25% degenerative spondylolisthesis of L5 on S1 with associated lower lumbar degenerative change. Previous conservative treatment, including medication, medial branch blocks, facet joint blocks, and a prior intradiscal procedure that provided only temporary relief, had failed to provide sustained improvement. Concordant pain provocation during the prior intradiscal procedure, followed by pain relief after lidocaine administration, supported a disc-related pain component. Because surgery was not feasible due to socioeconomic circumstances, she underwent intradiscal injection of 3 mL of Adinizer-processed MFAT into the L5-S1 disc. At baseline, the visual analog scale (VAS) score was 7 and the Oswestry Disability Index (ODI) was 36/45 (80%). Pain remained severe during the first month after treatment, but symptoms gradually improved thereafter. At three months, the ODI improved to 9/45 (20%) and the VAS score decreased to 2; these improvements were maintained at the two-year follow-up, when the ODI remained 9/45 (20%) and the VAS score further decreased to 1. Serial plain radiographs did not show a definite progression of listhesis on visual comparison. This single case documents sustained symptomatic improvement after intradiscal Adinizer-processed MFAT injection; however, causality cannot be established, and the findings should be considered hypothesis-generating. Further studies with controlled designs and objective structural assessment are needed.

## Introduction

Degenerative lumbar spondylolisthesis is a common cause of chronic low back pain and functional limitation in older adults. It is typically associated with intervertebral disc degeneration, facet arthropathy, and segmental instability, and may present with axial pain, morning stiffness, neurogenic symptoms, or activity-related mechanical back pain [1]. Initial treatment usually consists of conservative measures such as medication, physical therapy, bracing, and image-guided spinal injections [2, 3]. However, some patients continue to experience persistent symptoms despite these approaches, while surgical treatment may not always be feasible or acceptable.

In recent years, biologic therapies have been explored as potential minimally invasive options for chronic low back pain, particularly in patients with discogenic pain or degenerative disc disease. Prior studies of intradiscal biologic treatment have included autologous adipose-derived mesenchymal stem cells, bone marrow aspirate concentrate, and mesenchymal precursor or stromal cell-based therapies, with early reports suggesting acceptable safety and variable clinical improvement in selected patients [4-9]. However, the available evidence remains limited, heterogeneous, and focused primarily on discogenic low back pain rather than degenerative lumbar spondylolisthesis [4-9].

Among adipose-derived biologic products, minimally microfragmented adipose tissue (MFAT) can be prepared at the point of care using mechanical processing alone without enzymatic digestion. The Adinizer is a blade-based system designed to mechanically micronize lipoaspirate and generate injectable microfragmented adipose tissue [10, 11]. This distinction is clinically relevant because mechanically processed adipose tissue differs from enzymatically isolated stromal vascular fraction in both processing method and regulatory classification [10, 11]. Although mechanically processed MFAT has attracted increasing interest in regenerative medicine, literature specifically addressing intradiscal ADINizer-processed MFAT remains very limited.

Here, we report the two-year clinical course of a patient with symptomatic degenerative lumbar spondylolisthesis treated with intradiscal injection of Adinizer-processed MFAT after failure of prior conservative treatment and in a setting where surgery was not feasible. This case is presented to describe the procedural context and longitudinal clinical course of this approach. Given the single-case design, the findings should be interpreted as hypothesis-generating rather than as evidence of treatment efficacy.

## Case presentation

A 72-year-old woman presented with chronic low back pain. She reported that low back pain had been present for approximately six years but had worsened noticeably over the preceding six months. She described marked morning stiffness and pain aggravated by lumbar extension. Her most prominent associated symptom was bilateral buttock pain, which significantly interfered with daily activities. Prolonged standing and walking were limited because of pain, and her symptoms had progressively reduced her functional capacity.

On neurologic examination, sensation was intact and deep tendon reflexes were normal. Straight-leg-raise testing was positive at 30° bilaterally. Motor examination demonstrated pain-limited weakness, with bilateral hip flexion and ankle dorsiflexion graded as Medical Research Council grade IV. However, the patient did not report definite radicular pain, and her symptoms were dominated by low back and bilateral buttock pain rather than dermatomal radiation. In this context, the clinical presentation was considered more consistent with chronic mechanical low back pain with a disc-related pain component rather than definite lumbar radiculopathy. Because the patient’s pain radiated to both buttocks, alternative pain generators, including facet-mediated pain and sacroiliac joint-related pain, were also considered. However, facet loading tests and sacroiliac joint provocative maneuvers, including anterior gapping, posterior gapping, and thigh thrust tests, were negative. These findings reduced the likelihood that facet arthropathy or sacroiliac joint dysfunction was the primary pain source.

**Figure 1:**
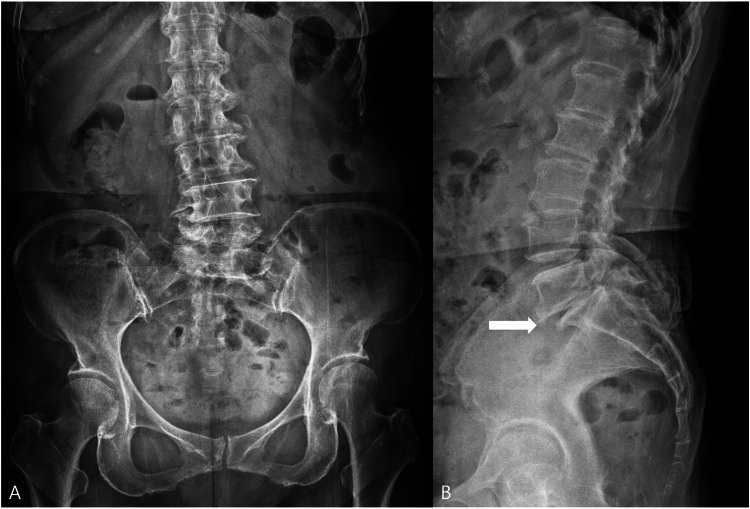
Baseline lumbar radiographs (A) Anteroposterior view of the lumbar spine showing degenerative change in the lower lumbar segments. (B) Lateral view demonstrating less than 25% degenerative spondylolisthesis of L5 on S1 with associated L5-S1 disc degeneration (white arrow).

Plain radiographs of the lumbar spine demonstrated less than 25% degenerative spondylolisthesis of L5 on S1, with associated lower lumbar degenerative change (Figure 1). Magnetic resonance imaging was not obtained because the patient did not have insurance coverage. The patient had longstanding symptoms, and the recent exacerbation had persisted for more than six months before the present intervention. The patient had experienced low back pain for approximately six years, with worsening over the preceding six months. The structured conservative treatment immediately preceding the present procedure included two weeks of medication, as well as two facet joint blocks and two medial branch blocks performed at two-week intervals; however, these measures did not provide sustained relief. She had also undergone a prior provocative intradiscal procedure, after which her buttock pain improved for approximately two weeks before recurring. During that procedure, concordant pain provocation reproduced the patient’s typical symptoms, and subsequent lidocaine administration resulted in symptom relief, supporting a disc-related pain generator. Based on the negative facet and sacroiliac provocative tests, the failure of prior facet-oriented interventions, and the temporary but concordant response to provocative intradiscal injection, the working diagnosis favored a disc-related pain generator. Because surgery was not feasible due to socioeconomic circumstances, the patient declined surgery and elected to undergo intradiscal treatment with MFAT.

**Figure 2:**
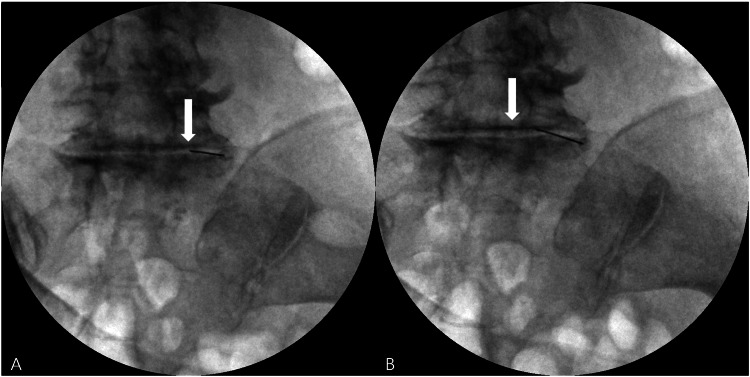
Fluoroscopic images obtained during intradiscal injection at L5-S1. (A) Needle position within the L5-S1 disc space under fluoroscopic guidance (white arrow); (B) Post-injection fluoroscopic image obtained after intradiscal administration at L5-S1 (white arrow).

At baseline, her visual analog scale (VAS) pain score was 7 and the Oswestry Disability Index (ODI) was 36/45 (80%), indicating severe disability [12]. Under sterile conditions, a tumescent solution was infiltrated into the lower abdomen, and adipose tissue was harvested under local anesthesia without sedation. A total of 60 mL of lipoaspirate was obtained. After washout, 10 mL of purified fat remained and was mechanically processed using the Adinizer, a blade-based system that micronizes adipose tissue without enzymatic digestion, to obtain MFAT. The total processing time was approximately 30 minutes. Skin preparation and draping were performed using povidone-iodine and sterile drapes, and prophylactic antibiotics were administered. Intradiscal injection was then performed under fluoroscopic guidance without additional sedation, and a total of 3 mL of MFAT was injected into the L5-S1 intervertebral disc (Figure 2). The injected volume was selected with reference to prior reports of lumbar intradiscal biologic injection using 3 mL of injectate per target disc [13].

**Figure 3:**
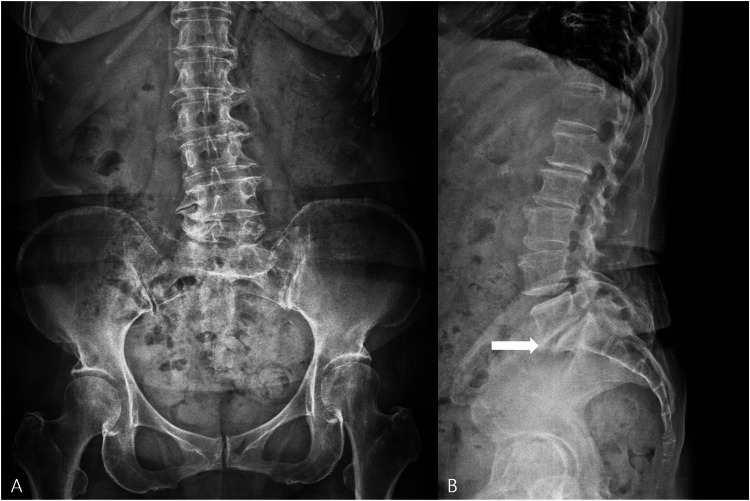
Follow-up lumbar radiographs obtained two years after treatment. (A) Anteroposterior view showing persistent lower lumbar degenerative change. (B) Lateral view demonstrating overall stable alignment without definite progression of L5-S1 degenerative listhesis on visual comparison (white arrow).

After the procedure, the patient wore a lumbar brace and underwent rehabilitation focused on pelvic floor muscle strengthening and lumbar isometric exercises. During the first month after treatment, pain remained severe, and she continued rehabilitation while wearing the brace and spending most of her time resting at home in a supine position. Her symptoms gradually improved thereafter. At three months, the ODI had improved to 9/45 (20%) and the VAS score had decreased to 2. At the two-year follow-up, the ODI remained 9/45 (20%) and the VAS score had further decreased to 1, indicating sustained improvement in both pain and disability. Serial lumbar radiographs obtained in March 2024 and March 2026 showed no definite radiographic progression of degenerative listhesis on visual comparison (Figure 3).

## Discussion

The main clinical significance of this case is that intradiscal injection of Adinizer-processed MFAT was applied in a patient with symptomatic degenerative lumbar spondylolisthesis who had longstanding low back pain, recent symptom aggravation, failure of multiple conservative interventions, and inability to proceed with surgery because of socioeconomic circumstances. In this context, the present report does not establish treatment efficacy but rather describes the procedural application and longitudinal clinical course of this approach in a selected patient with chronic mechanical low back pain and a disc-related pain component.

Most published intradiscal biologic studies for chronic low back pain have focused on discogenic pain and degenerative disc disease rather than degenerative lumbar spondylolisthesis [4-9]. Prior clinical reports have investigated intradiscal administration of adipose-derived mesenchymal stromal cells, bone marrow aspirate concentrate, and mesenchymal precursor or stromal cell-based therapies, with early studies suggesting acceptable safety and variable symptom improvement in selected patients [4-9]. However, the magnitude and consistency of clinical benefit remain heterogeneous, and efficacy has not been definitively established [4-9]. Against this background, the present case is notable not because it proves effectiveness, but because it extends this line of observation to a patient with degenerative lumbar spondylolisthesis rather than isolated discogenic low back pain.

An additional feature of this case is that the patient’s dominant associated symptom was bilateral buttock pain rather than definite radicular pain. Sensory examination and deep tendon reflexes were normal, and although straight-leg-raise testing was positive and pain-limited weakness was observed, the overall pattern was more consistent with chronic mechanical low back pain with a disc-related pain component than with clear root-specific lumbar radiculopathy. The prior intradiscal procedure is also clinically relevant: concordant pain provocation reproduced the patient’s typical symptoms, and her buttock pain temporarily improved after intradiscal treatment before recurring within two weeks. Although this sequence does not prove causality, it supported the working clinical impression that the disc contributed to her pain presentation and provided part of the rationale for proceeding with MFAT treatment.

Other minimally invasive intradiscal options, including intradiscal electrothermal therapy (IDET), may also be considered in selected patients with chronic disc-related low back pain. In the present case, however, a biologic intradiscal approach was favored because facet loading and sacroiliac provocative tests were negative, prior facet-oriented interventions had failed to provide sustained benefit, and provocative intradiscal injection reproduced the patient’s typical symptoms with temporary relief thereafter. Accordingly, this case was not intended to suggest the superiority of MFAT over IDET or other intradiscal procedures but rather to explain the clinical reasoning for selecting MFAT in this specific context.

The temporal pattern of recovery should be interpreted cautiously. During the first month after treatment, the patient continued to experience severe pain, required brace use, and spent much of her time resting at home in a supine position. Her symptoms improved gradually thereafter, with the ODI improving from 36/45 (80%) at baseline to 9/45 (20%) at three months and remaining at that level at two years, while the VAS score decreased from 7 at baseline to 2 at three months and to 1 at two years. Although this sustained improvement is clinically noteworthy, it cannot be attributed solely to intradiscal MFAT injection. Alternative explanations include the natural fluctuation of chronic low back pain, regression to the mean, placebo response associated with an invasive procedure, the effects of brace use and rehabilitation, and the combined influence of the overall treatment context. Accordingly, this case should be interpreted as hypothesis-generating rather than confirmatory.

Radiographically, serial lumbar radiographs did not show definite progression of degenerative listhesis on visual comparison over the follow-up interval. However, this observation should also be interpreted with caution. Visual comparison is inherently subjective, and apparent differences in disc space or alignment may partly reflect variation in positioning, sagittal posture, or projection. In addition, quantitative slip measurement, formal Meyerding grading over time, disc height measurement, and dynamic flexion-extension radiographs were not obtained, limiting objective structural assessment. Therefore, the imaging findings in this case should not be interpreted as evidence of structural regeneration or stabilization.

This report has several important limitations. First, it describes a single uncontrolled case and therefore cannot establish causality or generalizability. Second, there was no control comparator, no blinding, and no standardized observer-independent outcome assessment beyond patient-reported measures, which increases susceptibility to placebo effects and observer bias. Third, concurrent brace use and rehabilitation may have contributed to the observed improvement, making it difficult to isolate the specific effect of intradiscal MFAT injection. Fourth, advanced follow-up imaging, such as magnetic resonance imaging, was not available, and quantitative radiographic assessment of listhesis was not performed. Finally, although procedural details have been clarified, broader conclusions regarding optimal patient selection, dose, mechanism, and durability cannot be drawn from a single case.

Nevertheless, the favorable two-year clinical course in this patient suggests that further study of Adinizer-processed MFAT may be warranted in carefully selected patients with lumbar degenerative conditions, particularly in settings where standard conservative care has failed, and surgical treatment is not feasible or acceptable. Future studies should use controlled designs, clearer phenotypic characterization of pain generators, objective structural outcome measures, and standardized follow-up protocols to determine reproducibility and better define the role of this approach.

## Conclusions

In this single case, intradiscal injection of Adinizer-processed MFAT was followed by sustained improvement in pain and disability. However, the findings are hypothesis-generating only and do not establish treatment efficacy or structural benefit. Controlled studies with objective outcome measures are needed before clinical applicability can be determined.
